# Experimental Study and Application of Controlled Low-Strength Materials in Trench Backfilling in Suqian City, China

**DOI:** 10.3390/ma17040775

**Published:** 2024-02-06

**Authors:** Jingmin Xu, Qiwu Luo, Yong Tang, Zhibo Zeng, Jun Liao

**Affiliations:** 1 China Construction Fifth Engineering Division Corp., Ltd., Changsha 410019, China; 2 School of Transportation, Southeast University, Nanjing 211189, China

**Keywords:** trench backfilling, controlled low-strength materials (CLSM), flowability, compressive strength

## Abstract

When backfilling narrow spaces, controlled low-strength materials (CLSM) can be used to achieve an effective backfilling effect. The pipeline engineering in Yahnghe Avenue of Suqian, China, provides a favorable on-site condition for the use of CLSM. However, no guidance exists for the determination of the material mixture ratio of CLSM for this geological condition. Laboratory tests were performed to investigate the basic physical parameters of excavated soil and the optimal mixture ratio of CLSM. Results indicate that the sand and silt account for 29.76% and 57.23% of the weight of excavated soil, respectively. As the water content increases (from 40% to 50%), the flowability of the CLSM approximately shows a linear increase (slumps values from 154.3 mm to 269.75 mm for 9% cement content), while its compressive strength shows a linear decreasing trend (from 875.3 KPa to 468.3 KPa after curing for 28 days); as the cement content increases (from 6% to 12%), the flowability approximately shows a linear decreasing trend (from 238.8 mm to 178.5 mm for 45% water content), while the compressive strength shows a linear increasing trend (from 391.6 KPa to 987.6 KPa after curing for 28 days). By establishing the relationship between compressive strength/flowability and the water–cement ratio, the optimal material ratio is determined to be 9% cement content and 40–43% water content. The engineering application results indicate that the use of CLSM can achieve efficient and high-quality backfilling effects for pipeline trenches. The findings of this research may provide a reference for the application of CLSM in fields with similar geological conditions.

## 1. Introduction

In urban construction projects, the use of solid materials to backfill trenches is a common backfilling method. However, narrow spaces exist between excavation faces and structures (for instance, pipelines), and those spaces are difficult to backfill and compact. This can easily cause settlement and collapse during road operation, leading to the failure of the subgrade [1]. In addition, with traditional backfilling techniques it is also difficult to meet the requirements of rapid construction conditions and high backfilling quality [1]. 

To solve the above-mentioned engineering problems, a new type of fluidized cement–soil backfill technology known as the use of controlled low-strength materials (CLSM), is gradually being adopted in engineering [2]. According to the American Concrete Institute [3], CLSM is defined as a new backfilling material with high flowability, suitable compressive strength, and that can be self-compacted with less vibration under the action of self-weight. This technology mainly involves adding an appropriate amount (6–15%) of binding material (for instance, cement and fly ash), water, and/or curing agent to a specific soil mass, and they are mixed evenly to form a mixture with a certain fluidity. It is found that the CLSM adopts a higher proportion of cement (or fly ash) than other mixtures, such as the full-depth reclamation of pavements with cement [4], soil–cement [5] or cement-treated mixtures of RAP [6]. Then, the mixture is poured into the trench or other areas that need to be backfilled using a chute or pump. After solidification, a backfill body with a certain strength is formed. Therefore, the waste soil generated from pipeline excavation can be used to prepare CLSM on-site and then backfilled in the trench. This can simultaneously solve three engineering problems: soil treatment, procurement of filling material, and backfilling construction. The compressive strength and flowability of CLSM are important parameters [3]. Suitable flowability is crucial for self-compacting and self-leveling. The compressive strength in the early stage of curing should be sufficient for the resumption of traffic, and the final compressive strength should not be too high given the potential need for re-excavation in the future [7,8,9]. Therefore, it is of practical importance to study the key parameters governing the performance of CLSM and its application in engineering. 

Due to numerous advantages, the performance and application of CLSM have been investigated extensively. For instance, Kaneshiro et al. [9] explored the use of controlled low-strength material in the backfilling of a pipeline trench. The authors selected the particle size distribution, water-to-solid ratio and binder-to-aggregate ratio as the performance control parameters and experimentally assessed the effects of these factors on the physical and mechanical properties of CLSMs. The obtained optimal mixture ratio was tested in a field test, demonstrating the feasibility of the proposed CLSM for trench backfilling. Finney et al. [10] reported four case studies in Northern California where native soils were used exclusively to produce CLSM for pipeline backfilling. The authors discussed the effects of the differences between native soils on the performance of CLSM and designed a specific mixture ratio for each project. Based on 115 literature articles, Ling et al. [2] summarized that the materials used for the production of CLSM varied from case to case, which in turn has a significant impact on the performance of CLSM and its application in the field. Similarly, Dalal et al. [11] gave a detailed discussion on the recent advances in the development of CLSM with different waste materials, and the effects of material properties on the flowability, strength, and hardening time of CLSM. Kaliyavaradhan et al. [12] presented a review on the strength and excavatability of strength material CLSM, and highlighted the need to consider the supply, transport of materials, and material properties before being used in engineering practice.

In summary, it can be seen that there have been many studies on the application of CLSM, indicating its potential advantages in construction projects. Although Blanco et al. [13] proposed a preliminary methodology for the design of optimized CLSM combining FEM simulation and experimental testing, the design of CLSM is primarily based on experimental approaches [14,15,16,17,18,19]. For instance, Fauzi et al. [14] adopted laboratory testing and an empirical response surface method to evaluate the effect of key parameters on the properties of CLSM mixes produced with unprocessed fly ash and recycled fine aggregate. The authors proposed mix design models to reveal the relationships between the mixing parameters and the CLSM properties. A review by Ibrahim et al. [15] highlighted the importance of cement content, curing time, and temperature on the strength of CLSM based on experimental results. Nevertheless, no guidance was provided to determine a suitable mixture ratio. Kuo et al. [16] experimentally studied the hardened properties and the durability of CLSM mixed with waste oyster shells, cement, sand, and fine aggregate, suggesting that waste oyster shells are effective in the replacement of sand. Türkel [18] adopted direct shear testing to assess the shear strength of CLSM materials (a mixture of high-volume fly ash, crushed limestone powder, and pozzolana cement), and concluded that CLSM mixtures have excellent shear strength properties compared to compacted soil. Importantly, Wang et al. [19] conducted a comprehensive review of the mechanical properties of green CLSM and have summarized the effects of the binding material types and contents, water content, and admixture on the compressive strength of CLSM. Results demonstrate the complex interplay between those parameters and the properties of CLSM and the highly non-linear influence of key parameters on the compressive strength of CLSM. These results suggest that numerous factors affect the strength of CLSM. In other words, there is still no effective instruction to determine the optimized control parameters in specific engineering applications; for instance, Suqian Yanghe Avenue, China.

In Suqian City, China, the excavated soil for the drainage pipeline trench of the YHDD-TJ1 section of Yanghe Avenue is high moisture content silt soil, which is not suitable for direct trench backfilling. To utilize the excavated soils and save the cost of backfilling material procurement, the excavated silt soil was used to produce CLSM for backfilling pipeline trenches. To determine the mixture ratio of CLSM and to ensure safe backfilling, an experimental study and field testing were conducted. First, the physical properties of the excavated soil were tested. This was followed by the experimental investigation of the effects of key control parameters (cement content, water content, and curing time) on the performance (flowability and compressive strength) of CLSM in Yanghe Avenue. Then, key control parameters were optimized using the experimental data. Finally, the obtained CLSM technique was tested in Yanghe Avenue of Suqian City, achieving an efficient backfilling effect. This paper provides a unique dataset of the properties of the CLSM materials for the high moisture content silt soil, which is spread throughout the East China region. The results may be useful for the further application of CLSM technology under similar soil conditions.

## 2. Materials and Methods

**Figure 1:**
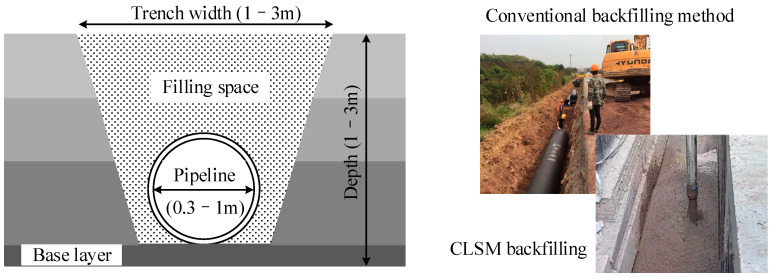
Illustration of the filling space and filling methods in the pipeline engineering.

The YHDD-TJ1 section of the Yanghe Avenue Reconstruction Project in Suqian City, China has a total length of 6.881 km, starting from S325 Provincial Road and ending in Kangcheng Road. This section of the drainage project involved a large number of open-cut pipeline construction activities. After the pipeline was installed, the closed water test was conducted, and then the trench backfilling was carried out, as shown in Figure 1. The on-site investigation results show that the excavated soil from the Yanghe Avenue project pipeline trench is high moisture content silty soil. If the trench was directly backfilled using the excavated soil, the backfilling effect in narrow spaces would be poor. Therefore, CLSM technology was used in this project. 

The performance of CLSM has a significant impact on the successful application of this technology. The flowability of CLSM is important. On the one hand, greater flowability can ensure a better filling effect in narrow spaces. On the other hand, the higher the water content, the lower the strength of the CLSM after curing. If the strength is lower than the threshold, it may not meet the engineering requirement for the strength of the subgrade [3]. Therefore, it is necessary to have a good balance between the water content and cement content to meet both strength and flowability requirements. Based on extensive field-testing experiences, the flowability of CLSM within the range of 150–200 mm can meet the requirements of pumping and achieve self-compacting filling in narrow spaces. The strength of CLSM after solidification and the time to reach its initial setting strength need to be determined according to specific engineering requirements. 

## 3. Experimental Design

### 3.1. Basic Physical Properties of the Excavated Soil

**Figure 2:**
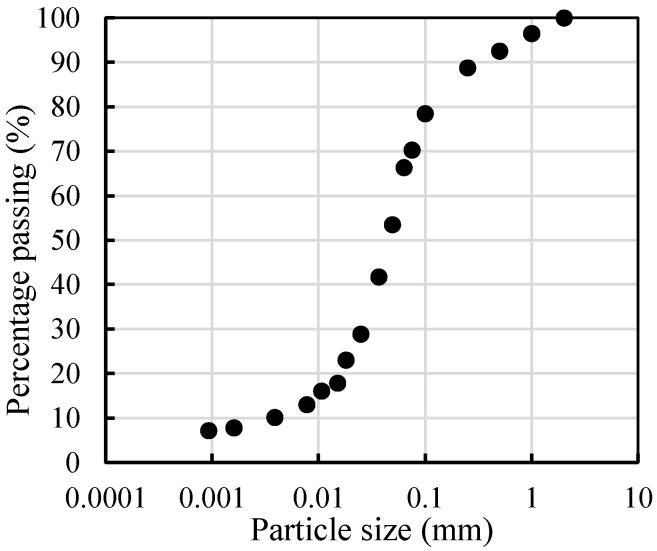
Particle size distribution curve.

The excavated silt soil in the typical road sections was selected and tested using a densimeter and laser particle analyzer Microtrac S3500 (Microtrac MRB, York, PA, USA) to obtain the grain composition of the silt soil. The basic physical properties, including organic matter content and water content, of the excavated soil were measured through weighing. The particle size distribution is shown in Figure 2 and Table 1.

Table 1 shows that the sand content of the soil sample is 29.76%, and the silt content is 57.23%. It is calculated that the non-uniformity coefficient Cu of the grading index is 16.35 and the curvature coefficient Cc is 41.79. These results meet the condition of Cu ≥ 5, but do not satisfy the condition of Cc = 1–3. According to “Regulations for Highway Geotechnical Testing” [20], the particle size distribution of the soil sample in this study is between silty soil and sandy soil. Based on the testing results, it is found that the organic matter content Wu of the excavated soil is 0.28%, which is much smaller than 5% (having negligible effects on the production of CLSM). According to the classification of the soil, it can be classified as inorganic soil. Finally, the experimental results also show that the average water content of the excavated soil is about 10%. 

### 3.2. Materials

To simplify the production procedure of CLSM, cement is used as the binding material. Therefore, water and cement were added to the excavated soil, and they were mixed evenly to prepare the CLSM. To investigate the effects of cement content, water content and curing time on the performance of the CLSM, the testing plan shown in Table 2 was designed. The cement content, which is defined as the ratio of the weight of cement to the weight of dry soil, is between 6% and 12%; water content, defined as the ratio of the weight of water to that of dry soil, is between 40% and 50%. The curing time of CLSM is set as 3 days, 7 days, and 28 days. Therefore, a total of 27 combinations were tested. 

### 3.3. Flowability Tests

**Figure 3:**
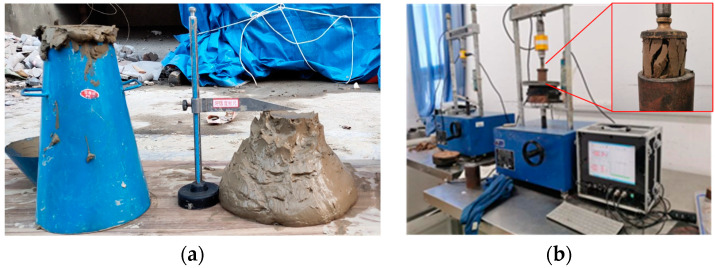
(**a**) Slump cone and (**b**) CBR-2 testing equipment with damaged specimen.

To test the flowability of the CLSM, the trumpet shaped slump cone (see Figure 3a) with an upper opening of 100 mm, a lower opening of 200 mm, and a height of 300 mm, was used, along with 1 measuring cylinder, 1 tamping rod, and 2 steel rulers, each with a measuring range exceeding 300 mm. The mixture of the CLSM was prepared according to Table 2 and three parallel experiments on each group of ratios were conducted. The average value was used as the final result of flowability. 

### 3.4. Compressive Strength Testing

The CLSM was prepared according to Table 2, and specimens were made using man-made molds. The unconfined compressive strength of the CLSM specimens was tested using CBR-2 California bearing ratio equipment, as shown in Figure 3b. The unconfined compressive strength of the sample is defined as *q*_u_, calculated as follows:

*q*_u_ = *F*/*A* (1)

where *q*_u_ is the unconfined compressive strength of the specimen (kPa); *F* is the maximum load at which the specimen fails (kN); and *A* is the cross-sectional area of the specimen (m^2^). 

In this paper, three parallel experiments on each group were performed and the average value was used. Therefore, a total of 27 × 3 = 81 samples were tested. 

## 4. Experimental Results

The average unconfined compressive strength values and flowability values are presented in Table 3. To evaluate the repeatability and credibility of the results, relative standard deviation (RSD) values were also calculated. Table 3 shows that RSD values for all the data are below 10%, which is acceptable for this type of experiment. 

### 4.1. Flowability of CLSM

**Figure 4:**
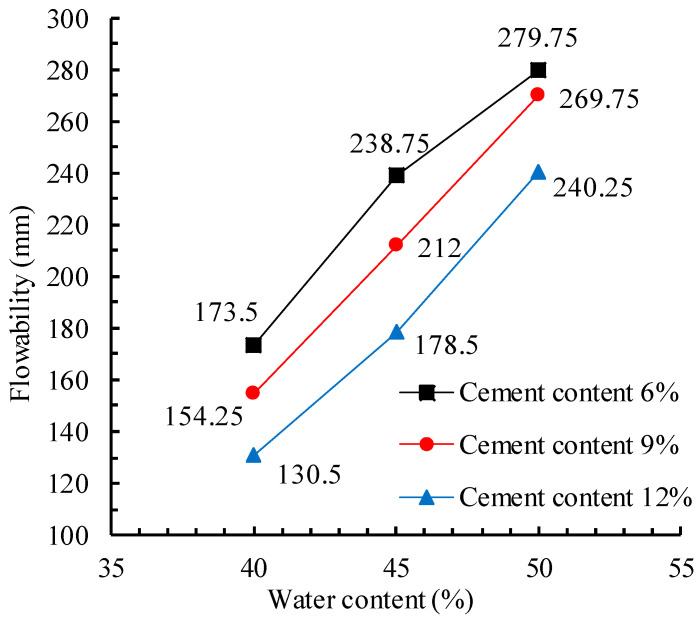
Flowability of the CLSM based on water content.

Figure 4 shows the variation of the flowability of the CLSM with respect to the water content. As the water content increases, the flowability of the CLSM approximately increases linearly. For instance, when the water content increases from 40% to 45%, the increase in flowability in all groups (6%, 9%, and 12% cement contents) of mixtures is about 38%; when the water content increases from 45% to 50%, the increments of flowability in three groups of mixtures are 18%, 27%, and 35%. This is because the dispersion effect of free water reduces the frictional resistance between solid particles and forms a lubricating water film that exists between solid particles. As the free water increases, the thickness of the water film also continuously increases, thus improving the flowability of the CLSM. 

When the cement content increases from 6% to 9%, the flowability of the CLSM decreases by about 11%. Among them, when the water content is 50%, the flowability value remains nearly unchanged when the cement content increases from 6% to 9%, indicating that there is sufficient water in the CLSM to undergo a hydration reaction with the cement. When the ash–soil ratio increases from 9% to 12%, the flowability values continuously decrease from 269.75 mm to 212 mm and then to 154.25 mm, with a decrease of 11.1%, 15.8%, and 15.4%, respectively. Those results broadly agree well with previous studies [2], which highlights the critical factors (water content and material properties) affecting the flowability of CLSM. 

### 4.2. Compressive Strength of CLSM

#### 4.2.1. Effects of Water Content

**Figure 5:**
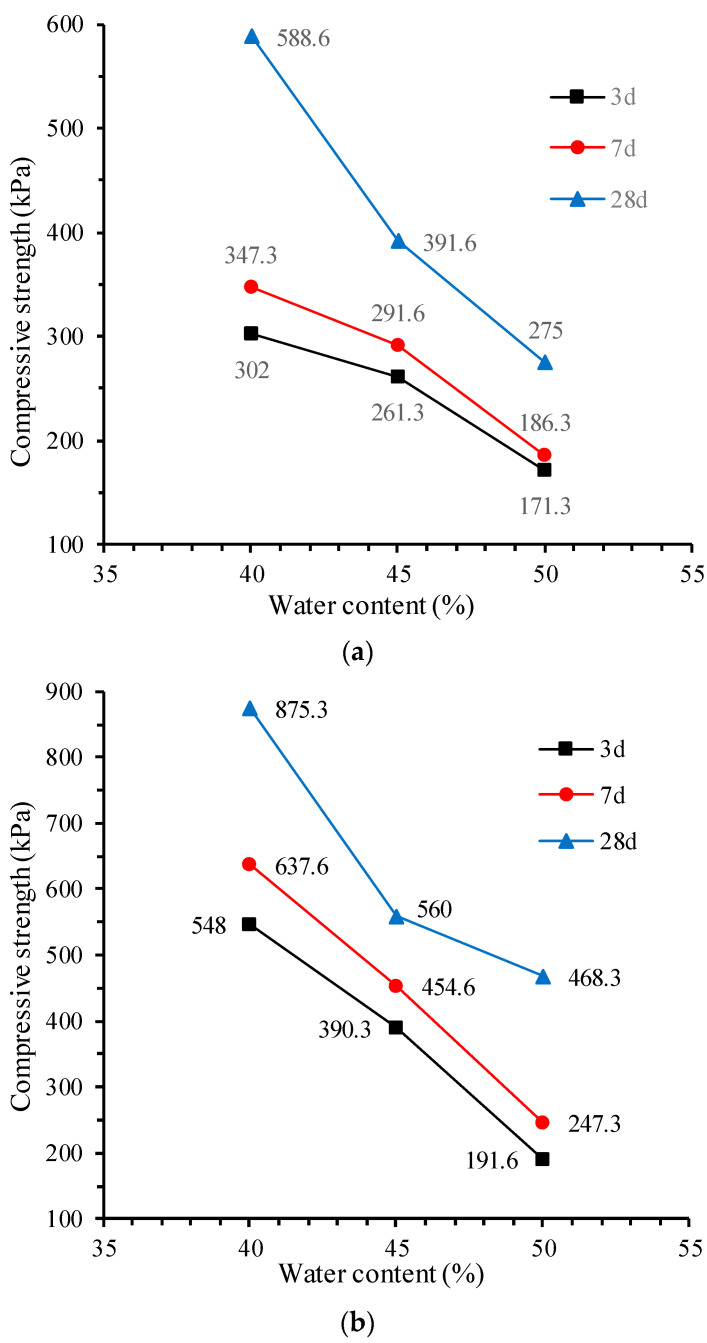

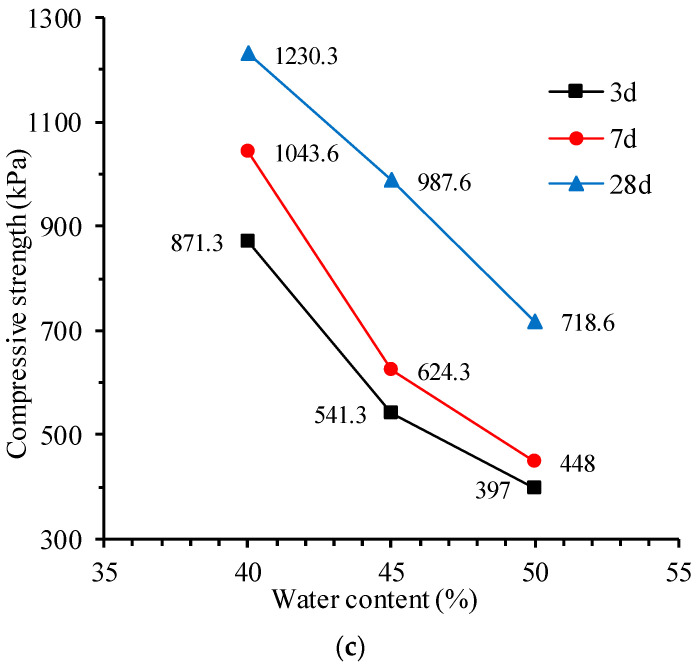
Influence of water content on the compressive strength of CLSM: (**a**) 6% cement content; (**b**) 9% cement content; and (**c**) 12% cement content.

Figure 5 shows the influence of water content on the unconfined compressive strength of CLSM. It is shown that when the water content increases from 40% to 50%, the compressive strength of the CLSM at different curing ages shows a linear decreasing trend, all by around 50%. When the cement content is 6%, the strength of CLSM decreases by 43.3% to 53.3%; when the water–cement ratio is 9%, the decrease in the compressive strength of the CLSM ranges from 46.5% to 65%; when the cement content is 12%, the decrease in strength ranges from 41.6% to 57%. Wang et al. [19] also reported the dominate role of the water–cement ratio in the compressive strength of CLSM, which broadly agrees with the results of this study. This aspect will be discussed in a later section. 

During the experiment, it was found that when the water content was 50%, the water would separate from the mixture during the static process of the sample. At the same time, some samples showed volume shrinkage after curing, indicating that a high water content may bring difficulties in evenly mixing the CLSM. Special attention should be paid during on-site construction.

#### 4.2.2. Effects of Cement Content

**Figure 6:**
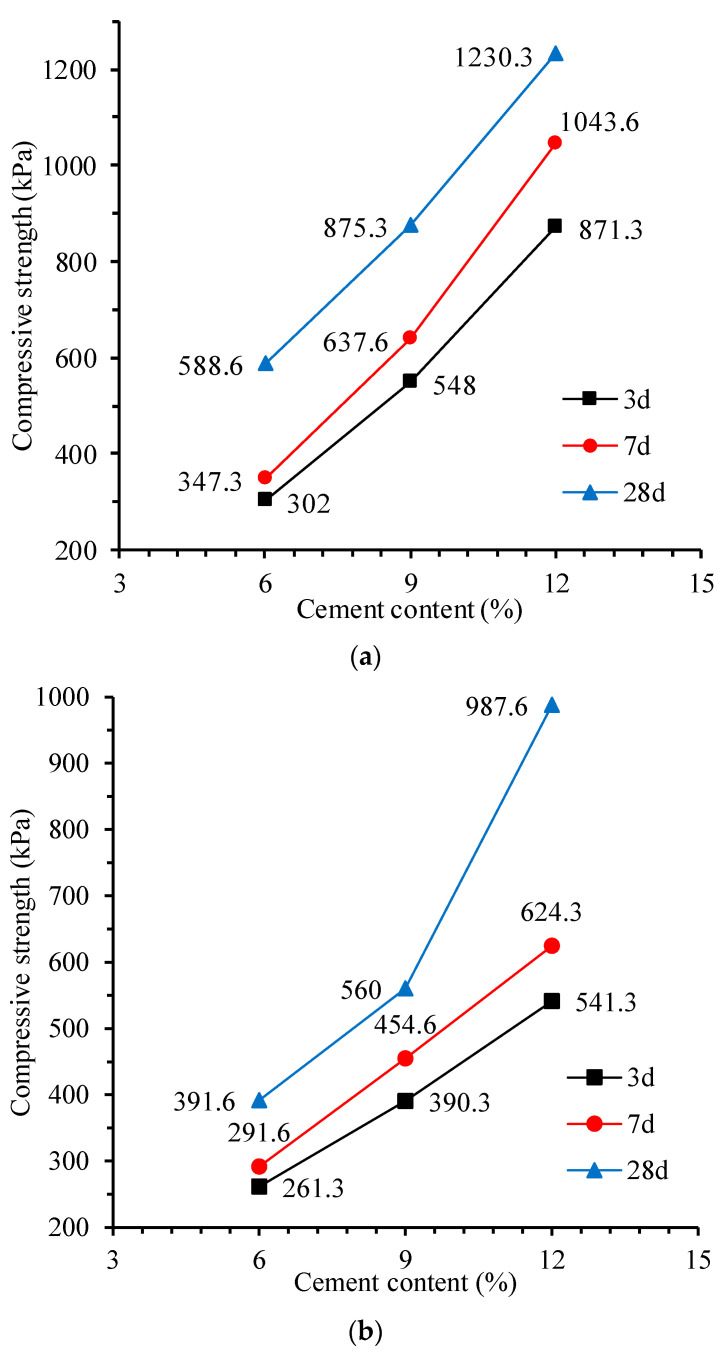

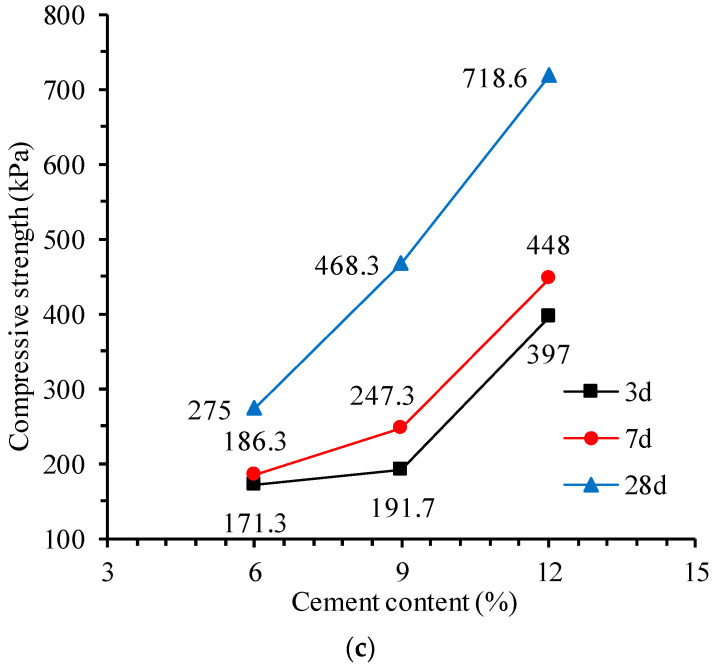
Influence of cement content on the compressive strength of CLSM: (**a**) 40% water content; (**b**) 45% water content; and (**c**) 50% water content.

Figure 6 shows the effect of cement content on the compressive strength of CLSM. It is shown that as the cement content increases, the compressive strength of CLSM samples at the given curing age continues to increase. Moreover, when the cement content increases from 9% to 12%, the strength of CLSM specimens increases significantly. This is because when the cement content is small, the degree of solidification of cementitious soil is low, and at this point, the overall cementitious skeleton structure has not yet formed within the mixture; with the increase in cement content, the hydration products also continue to increase, and the cementitious skeleton structure is densified, which improves the compressive strength of cementitious soil (CLSM) samples. Again, the results are in good agreement with Wang et al. [19], who summarized that the strength of CLSM increases linearly with the cement–aggregates ratio. 

#### 4.2.3. Effects of Curing Time 

**Figure 7:**
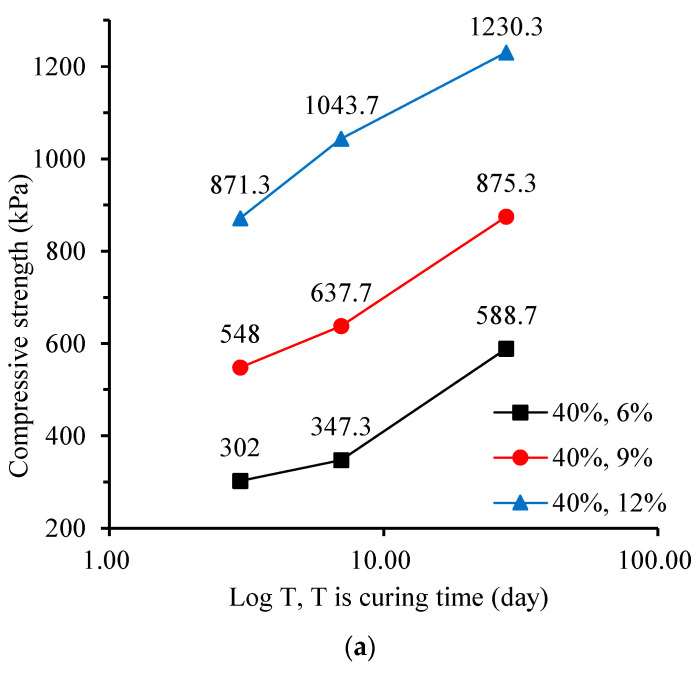

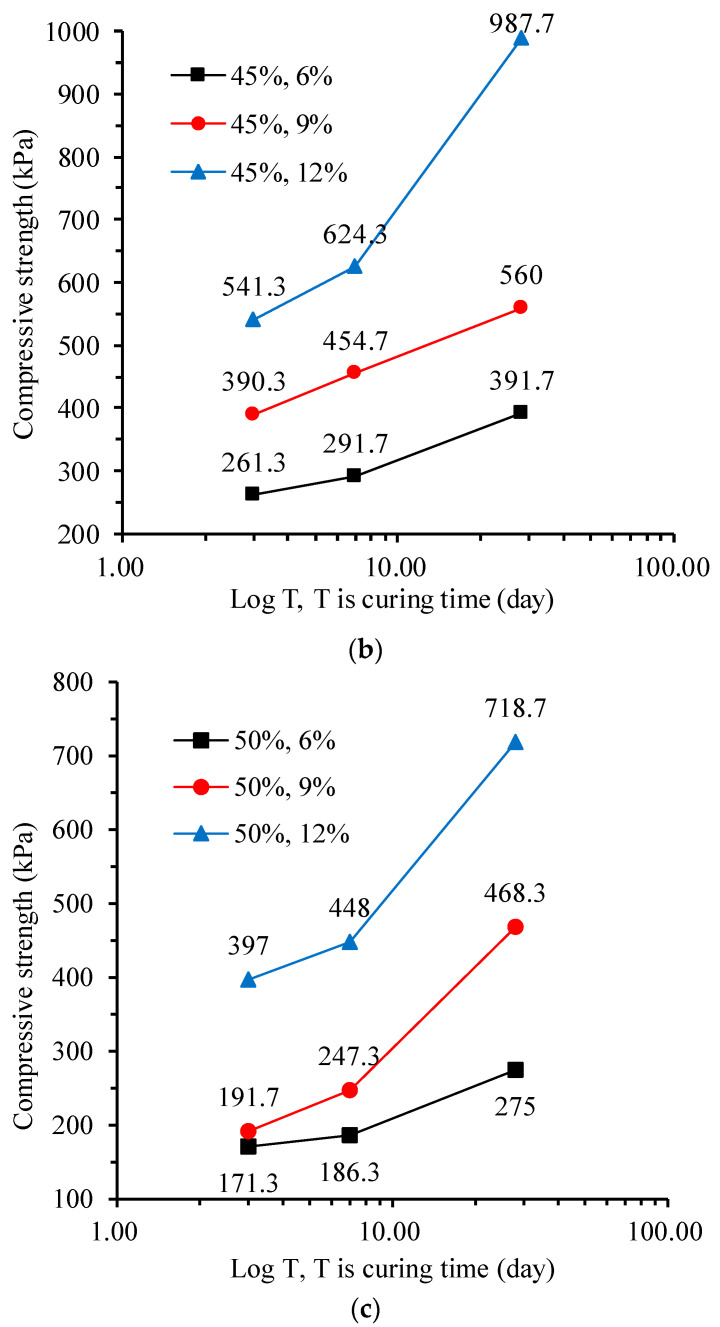
Influence of curing time on the compressive strength of CLSM: (**a**) 40% water content; (**b**) 45% water content; and (**c**) 50% water content.

Figure 7 shows the effect of curing time on the compressive strength of CLSM. As shown in Figure 7, the increase in compressive strength from 3 days to 7 days is limited, with a minimum increase of 8.76% and a maximum increase of 19.8%; the compressive strength increases significantly from 7 days to 28 days, with a minimum increase of 23.1% and a maximum increase of 69.5%. It is believed that the compressive strength of the CLSM sample increases approximately linearly with the logarithm of the curing time, as shown in Figure 7. As the curing time increases, the hydrolysis and hydration reaction of cement becomes more complete, increasing the number of hydration products and the strength of cementitious soil. In general, the compressive strength of cementitious soil will still increase after 28 days, and the trend of strength growth will gradually stabilize after exceeding 90 days [17,18,19]. 

### 4.3. Optimisation of the Ratio of Materials

**Figure 8:**
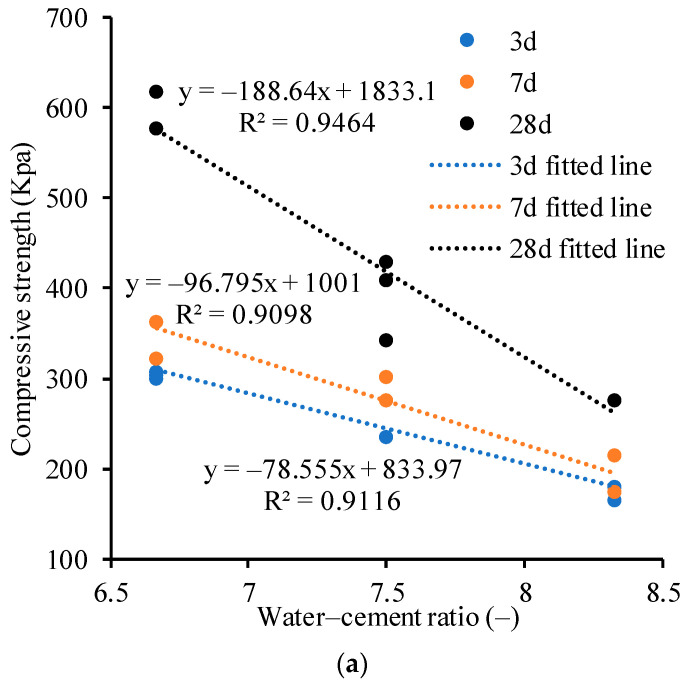

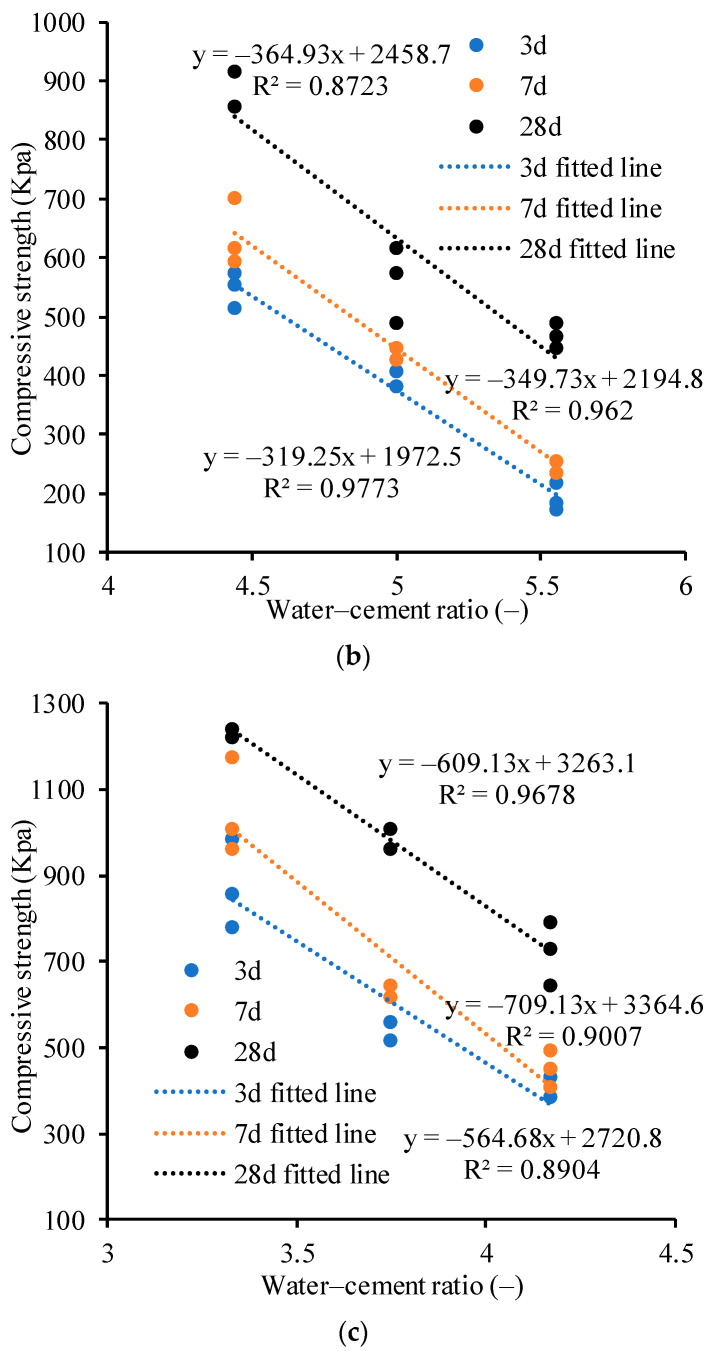
Influence of the water–cement ratio on the compressive strength of CLSM: (**a**) 6% cement content; (**b**) 9% cement content; and (**c**) 12% cement content.

The water–cement ratio refers to the ratio of the weight of water to that of cement. It is generally believed that the water–cement ratio is an important factor affecting the strength of cementitious soil [19]. Therefore, the compressive strength of CLSM is plotted against the water–cement ratio in Figure 8. Those results are the average values obtained from three parallel tests. Then, empirical equations can be established between the water–cement ratio and the unconfined compressive strength to determine the optimal material ratio of CLSM. 

Based on the experimental results, the fitting equations for the relationship between the compressive strength at 3 days, 7 days, and 28 days and the water–cement ratio were obtained, as shown in Figure 8. The obtained equations are as follows:

(1) For 6% cement content,
  *q*_u,3d_ = −78.555*m* + 833.97 (2)
  *q*_u,7d_ = −96.795*m* + 1001 (3)
  *q*_u,28d_ = −188.64*m* + 1833.1; (4)
(2) For 9% cement content,
  *q*_u,3d_ = −319.25*m* + 1972.5 (5)
  *q*_u,7d_ = −349.73*m* + 2194.8 (6)
  *q*_u,28d_ = −364.93*m* + 2458.7; (7)
(3) For 12% cement content,
  *q*_u,3d_ = −564.68*m* + 2720.8 (8)
  *q*_u,7d_ = −709.13*m* + 3364.6 (9)
  *q*_u,28d_ = −609.13*m* + 3263.1 (10)
  where *m* is the water–cement ratio and $q_{u,3d}$, $q_{u,7d},$ and $q_{u,28d}$ are the compressive strength of the CLSM in the curing time of 3 days, 7 days, and 28 days, respectively. The linear relationship between the compressive strength and the water–cement ratio is broadly in good agreement with previous studies on CLSM summarized by Wang et al. [19]. However, it is worth noting that the above equations were obtained from the experimental data of the excavated soil in Suqian City with the water content between 40% and 50%. Therefore, care should be taken when using these equations in other cases.

**Figure 9:**
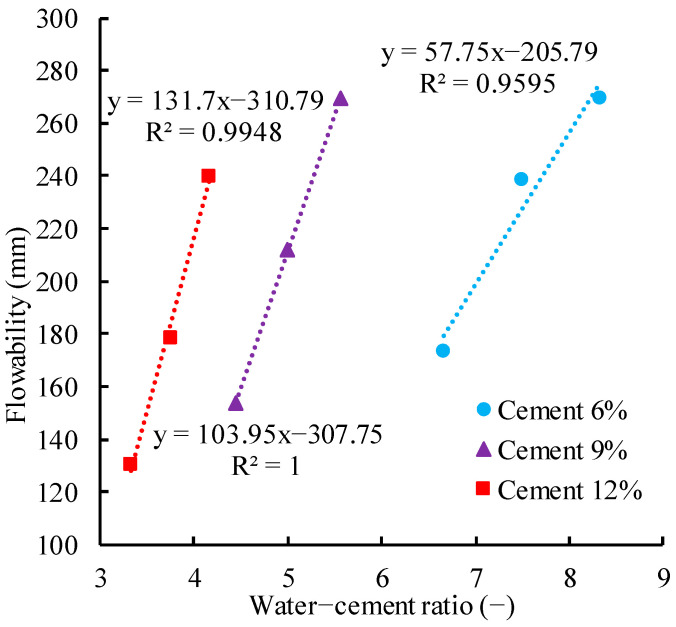
Relationship between water–cement ratio and flowability of CLSM.

Next, on the premise of meeting the compressive strength requirements, the flowability of CLSM also needs to satisfy the engineering requirements. By establishing the relationship between the water–cement ratio and flowability, as shown in Figure 9, the equations for 6%, 9%, and 12% cement content were obtained:

*f*_6_ = 57.89*m* − 206.78 (11)

*f*_9_ = 103.49*m* − 307.75 (12)

*f*_12_ = 130.65*m* − 306.87 (13)

where $f_{6}$, $f_{9},$ and $f_{12}$ are the flowability values (in mm) at 6%, 9%, and 12% cement content. 

According to the requirements of pipeline-trench backfilling engineering, combined with existing literature research and national standards [3,20], it is determined that the flowability of the CLSM should be within the range of 150 to 200 mm, and the unconfined compressive strength values at 3, 7, and 28 days should be no less than 200, 400, and 800 KPa. 

Based on the above analysis and Equations, it is concluded that when the cement content is 9%, the water–cement ratio should be within the range of 4.40~4.8 (equivalent to the water content within the range of 39.6~43.2%). These parameters guarantee both the compressive strength and flowability of the CLSM to meet the engineering requirements. Furthermore, for the cement content of 12% and the water–cement ratio within the range of 3.5~3.88 (equivalent to the water content within the range of 42~46.6%), both the compressive strength and flowability of CLSM can also satisfy the engineering requirements. In order to reduce the amount of cement and to save engineering costs, a 9% cement content and a 40–43% water content were adopted in the Yanghe Avenue pipeline trench backfilling project. 

## 5. Engineering Practice and Discussion

### 5.1. Preparation of and Backfilling with CLSM

**Figure 10:**
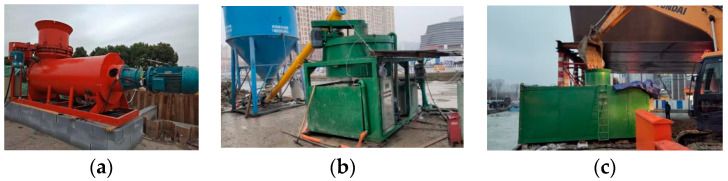
Equipment for the preparation and transportation of CLSM: (**a**) Soil metering device; (**b**) automated cement slurry preparation system; (**c**) intelligent CLSM mixing systems.

The pipeline-trench backfilling project in the Yanghe Avenue (YHDD-TJ1 section) drainage engineering construction adopted the CLSM backfilling technology for trench backfilling. The CLSM was produced based on the material mixture ratio obtained from the experiments (9% cement content and 40–43% water content). Based on the strength and flowability characteristics of the CLSM, the equipment suitable for mixing and backfilling was selected, as shown in Figure 10. 

The construction equipment in the field mainly includes the following systems: (1) an automated cement slurry preparation system, which can achieve automatic and accurate weighing, automatic feeding according to the material proportion, and high-speed and efficient mixing and slurry production through settings; (2) the intelligent CLSM mixing system allows for a continuous feeding and mixing of materials. The soil filling speed can be adjusted through a frequency converter for various soil filling speeds; (3) the excavator is used to fill the soil, and stones with a diameter greater than 100 mm need to be removed; and (4) transportation equipment, which is mainly suitable for short distance pumping and pouring. Importantly, the automated cement-slurry preparation and intelligent CLSM mixing systems are crucial for the uniformity of the CLSM and backfilling quality. Those systems/facilities were specifically designed and manufactured for the preparation of CLSM and backfilling narrow spaces. 

**Figure 11:**
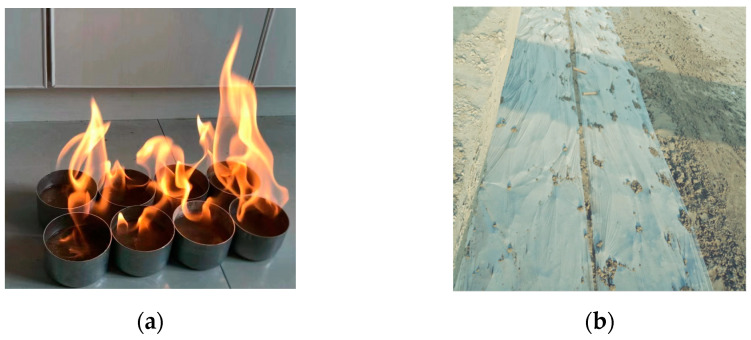
(**a**) Alcohol combustion method and (**b**) CLSM covered with plastic film.

The main construction process can be summarized as follows: (1) preparation of raw materials, including determination of soil water content (alcohol combustion method, see Figure 11a), and sorting of debris such as stones; (2) flowability determination, which includes testing the flowability based on the optimal ratio combined with the natural water content of the soil material; (3) adjustment of the construction parameters of the mixing system, mainly by adjusting the frequency converter to control the filling speed of the cement slurry and soil materials; (4) cement soil mixing to form the CLSM; (5) the CLSM was transported to the site using a tubular chute, filling the trench under self-weight; (6) because the depth of the trench is around 1 m, trench backfilling was completed at one time point; and (7) the CLSM was levelled and immediately covered with plastic film after initial setting, as shown in Figure 11b. 

### 5.2. Quality Detection 

To test the construction quality and backfilling effect, samples were obtained by drilling and coring, and the strength of the samples was tested. The total length for sampling was 200 m, and five groups of samples were taken, with an interval of 50 m; three samples were taken from each group along the middle and boundary of the trench. The unconfined compressive strength of the samples after curing 3 days, 7 days, and 28 days was tested using CBR-2 testing equipment. 

Experimental results show that the compressive strength values of the CLSM samples after 3 days, 7 days, and 28 days are all above 200, 400, and 800 KPa, respectively, and these results are in good agreement with the prediction of the proposed equations in Figure 8, indicating that the backfilling at different positions of the trench is relatively uniform and the strength of CLSM can fully meet the requirements of road engineering. The use of CLSM backfilling technology has achieved good backfilling effects for the trench backfilling in the YHDD-TJ1 section of the Yanghe Avenue Reconstruction Project in Suqian City, China. 

### 5.3. Discussion

This study presents an application of CLSM in the trench backfilling project of Suqian, Yanghe Avenue, China. Cement was used as the only binding material, and the flowability and the comprehensive strength of CLSM were experimentally investigated. An optimal material mixture ratio was obtained, and the application result is satisfactory. The findings in this research may be useful for the trench backfilling community. However, in this study, limited samples were tested and only the compressive strength, flowability, and curing time were considered. The properties of CLSM were not exhaustively analyzed. Future work could consider the shear strength, porosity sorptivity, durability (dry–wet cycles, freeze–thaw cycles, and chemical corrosion), microstructure evolution, and chemical reaction mechanism. 

## 6. Conclusions

This paper has presented an experimental and in situ testing study on the application of CLSM technology in the trench backfilling of the Yanghe Avenue of Suqian City, China. Cement was selected as the binding material. Laboratory tests and theoretical analysis were used to study the basic physical parameters of the excavated soil and the optimal material ratio of the CLSM. The backfilling quality of the trench in Yanghe Avenue of Suqian City, China, was also tested. Limitations are discussed and potential future work is suggested. 

The flowability and unconfined compressive strength of CLSM are the most important properties. Experimental results show that they are strongly affected by water and cement content: as the water content increases, the flowability value of CLSM shows an approximately linear increase, while the unconfined compressive strength shows a linear decreasing trend; as the cement content increases, the flowability value of CLSM shows an approximately linear decrease, while the compressive strength linearly increases (for the same curing age). These results agree well with previous studies [21,22,23]. 

The relationships between the water–cement ratio of CLSM and the unconfined compressive strength/flowability were obtained. Based on the experimental data, the mixture ratio for producing CLSM in Suqian Yanghe Avenue was optimized. The results show that using 9% cement content and 40–43% water content can meet the requirement of both compressive strength and flowability of the CLSM. These parameters can also help save cement consumption and reduce construction costs. 

The CLSM technique was used to backfill the pipeline trenches in the drainage project of Yanghe Avenue of Suqian, China. The performance of CLSM was evaluated by drilling and coring at a given curing time. The compressive strength of the specimens was tested using CBR-2 California bearing ratio equipment. The results show that the strength of CLSM samples can fully meet the requirements of road engineering, achieving good backfilling results. The obtained material mixture ratio of CLSM can also be used in other projects with similar soil conditions. 

## Figures and Tables

**Table 1:** Particle size distribution of silty sand.

**Particle (mm)**	>0.25	0.25~0.075	0.075~0.005	<0.005
**Percentage (%)**	11.16	18.6	57.23	13.01

**Table 2:** Cement soil flow proportioning scheme.

Cement Content (%)	Water Content (%)	Curing Time (%)
6	40, 45, 50	3, 7, 28
9	40, 45, 50	3, 7, 28
12	40, 45, 50	3, 7, 28

**Table 3:** Experimental results for unconfined compressive strength, flowability, and RSD.

Water (%)	Cement (%)	3 Days	7 Days	28 Days	Flowability
		*q*_u_ (KPa), RSD (%)	*q*_u_ (KPa), RSD (%)	*q*_u_ (KPa), RSD (%)	Value (mm), RSD (%)
40	6	302, 1.00	347.3, 6.81	588.6, 4.02	173.5, 6.56
	9	548, 5.67	637.6, 8.89	875.3, 4.02	154.25, 4.31
	12	871.3, 9.98	1043.6, 9.79	1230.3, 0.94	130.5, 4.74
45	6	261.3, 9.06	291.6, 4.95	391.6, 9.49	238.75, 1.05
	9	390.3, 3.70	454.6, 6.94	560, 9.57	212, 4.74
	12	541.3, 4.37	624.3, 2.31	987.6, 2.69	178.5, 6.51
50	6	171.3, 4.46	186.3, 10.39	275, 1.08	279.75, 1.02
	9	191.7, 10.62	247.3, 4.67	468.3, 4.38	269.75, 1.30
	12	397, 6.54	448, 9.15	718.6, 10.33	240.25, 1.04

## Data Availability

Data are contained within the article.

## References

[1] Prum S., Jumnongpol N., Eamchotchawalit C., Kantiwattanakul P., Sooksatra V., Jarearnsiri T., Passananon S. Guideline for Backfill Material Improvement for Water Supply Pipeline Construction on Bangkok Clay, Thailand. Proceedings of the 4th World Congress on Civil, Structural, and Environmental Engineering (CSEE’19).

[2] Ling T.C., Kaliyavaradhan S.K., Poon C.S. (2018). Global perspective on application of controlled low-strength material (CLSM) for trench backfilling–An overview. Constr. Build. Mater..

[3] American Concrete Institute (2005). ACI-229R, Controlled Low Strength Materials (Reproved 2005).

[4] Fedrigo W., Nunez W.P., Visser A.T. (2020). A review of full-depth reclamation of pavements with Portland cement: Brazil and abroad. Constr. Build. Mater..

[5] Linares-Unamunzaga A., Pérez-Acebo H., Rojo M., Gonzalo-Orden H. (2019). Flexural strength prediction models for soil–cement from unconfined compressive strength at seven days. Materials.

[6] Fedrigo W., Núñez W.P., López M.A.C., Kleinert T.R., Ceratti J.A.P. (2018). A study on the resilient modulus of cement-treated mixtures of RAP and aggregates using indirect tensile, triaxial and flexural tests. Constr. Build. Mater..

[7] Naik T.R., Singh S.S. (1997). Permeability of flowable slurry materials containing foundry sand and fly ash. J. Geotech. Geoenviron. Eng..

[8] Dockter B.A., Howard A.K., Hitch J.L. (1998). Comparison of dry scrubber and class c fly ash in controlled lowstrength materials (CLSM) applications. The Design and Application of Controlled Low-Strength Materials (Flowable Fill), ASTM STP 1331.

[9] Kaneshiro J., Navin S., Wendel L., Snowden H. (2001). Controlled low strength material for pipeline backfill—Specifications, case histories and lessons learned. Pipelines 2001: Advances in Pipelines Engineering and Construction.

[10] Finney A.J., Shorey E.F., Anderson J. (2008). Use of native soil in place of aggregate in controlled low strength material (CLSM). Pipelines 2008: Pipeline Asset Management: Maximizing Performance of our Pipeline Infrastructure.

[11] Dalal P.H., Patil M., Iyer K.K., Dave T.N. (2023). Sustainable controlled low strength material from waste materials for infrastructure applications: State-of-the-art. J. Environ. Manag..

[12] Kaliyavaradhan S.K., Ling T.C., Guo M.Z. (2022). Upcycling of wastes for sustainable controlled low-strength material: A review on strength and excavatability. Environ. Sci. Pollut. Res..

[13] Blanco A., Pujadas P., Cavalaro S.H.P., Aguado A. (2014). Methodology for the design of controlled low-strength materials. Appl. Backfill Narrow Trenches. Constr. Build. Mater..

[14] Fauzi M.A., Arshad M.F., Nor N.M., Ghazali E. (2022). Modeling and optimization of properties for unprocessed-fly ash (u-FA) controlled low-strength material as backfill materials. Clean. Eng. Technol..

[15] Ibrahim M., Rahman M.K., Najamuddin S.K., Alhelal Z.S., Acero C.E. (2022). A review on utilization of industrial by-products in the production of controlled low strength materials and factors influencing the properties. Constr. Build. Mater..

[16] Kuo W.T., Wang H.Y., Shu C.Y., Su D.S. (2013). Engineering properties of controlled low-strength materials containing waste oyster shells. Constr. Build. Mater..

[17] Liu H., Xiao Y., Liu K., Zhu Y., Zhang P. (2022). Numerical simulation on backfilling of buried pipes using controlled low strength materials. Appl. Sci..

[18] Türkel S.E.L.Ç.U.K. (2007). Strength properties of fly ash based controlled low strength materials. J. Hazard. Mater..

[19] Wang C., Li Y., Wen P., Zeng W., Wang X. (2023). A comprehensive review on mechanical properties of green controlled low strength materials. Constr. Build. Mater..

[20] (2020). China Standard: Regulations for Highway Geotechnical Testing.

[21] Khadka S.D., Okuyucu O., Jayawickrama P.W., Senadheera S. (2023). Controlled low strength materials (CLSM) activated with alkaline solution: Flowability, setting time and microstructural characteristics. Case Stud. Constr. Mater..

[22] Wan X., Ding J., Jiao N., Zhang S., Wang J., Guo C. (2023). Preparing controlled low strength materials (CLSM) using excavated waste soils with polycarboxylate superplasticizer. Environ. Earth Sci..

[23] Tran T.Q., Kim Y.S., Dang L.C., Do T.M. (2023). A state-of-the-art review on the utilization of new green binders in the production of controlled low-strength materials. Constr. Build. Mater..

